# Determination of the Dissolution/Permeation and Apparent Solubility for Microencapsulated Emamectin Benzoate Using In Vitro and Ex Vivo *Salmo salar* Intestine Membranes

**DOI:** 10.3390/ph15060652

**Published:** 2022-05-25

**Authors:** Victoria Molina, Carlos von Plessing, Alex Romero, Sergio Benavides, José Miguel Troncoso, José Ricardo Pérez-Correa, Wendy Franco

**Affiliations:** 1 Department of Chemical and Bioprocess Engineering, Pontificia Universidad Católica de Chile, Santiago 6904411, Chile; lvmolina@uc.cl (V.M.); perez@ing.puc.cl (J.R.P.-C.); 2 Faculty of Pharmacy, Universidad de Concepción, Concepción 4030000, Chile; cvonples@udec.cl; 3 Laboratory of Immunology and Stress of Aquatic Organisms, Animal Pathology Institute, Universidad Austral de Chile, Valdivia 5090000, Chile; alexromero@uach.cl; 4 Interdisciplinary Center for Aquaculture Research (INCAR), Centro FONDAP, Valdivia 5090000, Chile; 5 Research Center in Agri-Food and Applied Nutrition, Universidad Adventista de Chile, Chillán 3820572, Chile; sfbenavides@uc.cl; 6 Faculty of Sciences for Health Care, Universidad San Sebastián, Concepción 4080871, Chile; 7 Cargill Innovation Center, Cargill, Calbuco 5570130, Chile; jose_troncoso@cargill.com; 8 Department of Health Sciences, Nutrition and Dietetics Career, Pontificia Universidad Católica de Chile, Santiago 6904411, Chile

**Keywords:** emamectin benzoate, dissolution/permeation, release kinetics, apparent solubility, apparent permeability, uptake, intestine, *Salmo salar*

## Abstract

In this work, two microencapsulation techniques were used to protect and improve the absorption of emamectin benzoate (EB), which is an antiparasitic drug used to control *Caligus rogercresseyi*. EB has a low aqueous solubility, which affects its absorption in the intestine of *Salmo* *salar*. Microparticles were produced by spray drying and ionic gelation, using Soluplus^®^ (EB–SOL) and sodium alginate (EB–ALG) as polymers, respectively. Studies were conducted on dissolution/permeation, apparent permeability (Papp), apparent solubility (Sapp), and absorption using synthetic and biological membranes. Based on these results, the amount of EB in the microparticles needed to achieve a therapeutic dose was estimated. The EB–ALG microparticles outperformed both EB–SOL and free EB, for all parameters analyzed. The results show values of 0.45 mg/mL (80.2%) for dissolution/permeation, a Papp of 6.2 mg/mL in RS–L, an absorption of 7.3% in RS, and a Sapp of 53.1% in EM medium. The EB–ALG microparticles decrease the therapeutic dose necessary to control the parasite, with values of 3.0^−2^ mg/mL and 1.1^−2^ mg/mL for EB in EM and RS, respectively. The Korsmeyer–Peppas kinetic model was the best model to fit the EB–ALG and EB–SOL dissolution/permeation experiments. In addition, some of our experimental results using synthetic membranes are similar to those obtained with biological membranes, which suggests that, for some parameters, it is possible to replace biological membranes with synthetic membranes. The encapsulation of EB by ionic gelation shows it is a promising formulation to increase the absorption of the poorly soluble drug. In contrast, the spray-dried microparticles produced using Soluplus^®^ result in even less dissolution/permeation than free EB, so the technique cannot be used to improve the solubility of EB.

## 1. Introduction

The intestinal absorption of a drug is defined as the amount of the drug solubilized in the intestinal fluids that permeates through the membrane and reaches the bloodstream [1]. This absorption is primally regulated by two factors: (i) the solubility and dissolution characteristics in the gastrointestinal milieu, and (ii) the effective permeability across the intestinal mucosa and membrane [1]. Since the drug has to dissolve in the intestinal tract to be absorbed, compounds with low aqueous solubility are often poorly absorbed and, therefore, are less bioavailable than soluble ones.

Solubility is the maximum amount of a crystalline solute that can be dissolved in a solvent at a given temperature and pressure to form a homogeneous solution [2], and it is proportional to the rate at which the drug dissolves from the solid state (dissolution). The higher the solubility, the faster the dissolution [3]. Drugs with low solubility frequently present problems concerning their formulation and bioavailability. Drugs, in general, must be in a molecularly dispersed form (i.e., in solution) before they can be absorbed across biological membranes. In a passive membrane transport, the relative solubility of the drug in an aqueous medium and the lipid cell membrane is essential [3]. In drug delivery studies, solubility is also measured through the apparent solubility (Sapp), especially when there is an incomplete dissolution, or insufficient time to reach saturation [4], which is common in molecules with poor solubility. As well as solubility, Sapp depends on temperature, pH, type of solvent, and presence of salts or oils [5].

On the other hand, the interaction between the dissolved drug and the membrane defines the amount of drug that crosses the membrane; this process is measured as the apparent permeability (Papp) [6]. The permeability depends on the nature of the membrane (hydrophilicity or lipophilicity), its molecular weight, and polarity [7].

The lipophilicity of a compound is a physicochemical property, consisting of a hydrophobic effect and solute–solvent interactions that contribute to the distribution of a solute between two media: water and organic solvents, commonly expressed in terms of the octanol/water partition coefficient (Kow). This property is one of the driving forces for the passive transport of drugs across membranes [8].

A drug with low solubility, used by the Chilean salmon farming industry, is emamectin benzoate (EB) [9]. This drug is an antiparasitic used to control *Caligus rogercresseyi*, a pathogen that causes significant economic losses in Chilean salmon farming, mainly linked to treatment costs, decreases in the fish growth rate, and increased susceptibility to other diseases [9]. The emamectin benzoate treatment in salmon is administered through feed [9]. Therefore, adequate intestinal absorption of this antiparasitic is essential to improve its efficacy against *C. rogercresseyi* [9]. This drug is barely absorbed at the intestine, since it is highly water-insoluble at the pH of the intestine of *Salmo salar* (between 7 and 8) [10]. The aqueous solubility of EB decreases with pH. At pH = 5, S = 320 mg/L, while at pH = 7, S = 24 mg/L [9].

Additionally, EB has a crystalline structure, which is one of the causes of its low dissolubility. One alternative to increase dissolution is to embed the drug into a polymer matrix, simultaneously obtaining an amorphous structure and creating hydrophilic bonds [11]. This technique allows kinetic stabilization and immobilizes supersaturated EB concentrations, preventing phase separation and re-crystallization [12]. The structure is relevant for the drug’s release and absorption because both depend on the dissolution of the drug; if dissolution is low, there is a poor release and absorption. According to the Noyes–Whitney model, an amorphous structure has a higher drug surface area than a crystalline structure, which increases the dissolution rate [13].

Microencapsulation is an alternative that helps overcome the solubility and permeation problems associated with the administration of poorly soluble drugs. Microencapsulation techniques, in particular ionic gelation and spray drying, are typically used to improve the solubility of chemical compounds with low solubility and permeation [14,15].

However, problems related to loading and release, such as low drug concentrations at the active site and very short drug residence time at cellular and anatomical sites, were reported [16]. One of the most common problems in drug loading is the limited interaction capacity between the drug and the polymer [17]. In the case of release, one of the most common problems is the burst effect on drug release [18]. Both problems are related to the type of polymer. To overcome them, the ratio of drug to polymer, the molecular weight of the drug, and the composition of the polymer are considered.

In general, polymers respond to a stimulus or environment, such as changes in temperature, pH, light, and redox potential, inducing dynamic and reversible changes useful for releasing drugs [16]. These characteristics make the type of polymer crucial in drug load and release. Moreover, they must overcome anatomical and physiological barriers and administer drugs locally in the sites of interest, thus, improving therapy. The current trend is to find polymers that increase the efficacy while decreasing the toxicity of the drug [16].

The polymer used for the encapsulation largely determines the extent at which the drug stabilizes, and, therefore, influences its solubility and later permeation. In addition, the polymer used for encapsulation controls the release of the drug following the established and controllable kinetics [19]. Mathematical models commonly used to characterize the release kinetics obtained from dissolution/permeation are: (i) zero-order kinetics, (ii) first-order kinetics, (iii) the Higuchi model, (iv) the Ritger–Peppas and Korsmeyer–Peppas models, (v) the Weibull model, and (vi) the Peppas–Sahlin model [20]. The model defines the mechanism at which the encapsulated drug is released; therefore, it is common to select the encapsulating polymers in order to allow for a more efficient therapeutic effect at the desired time, rate, dose, and site of action [21]. Previous research shows that alginate and Soluplus^®^ (Ludwigshafen, Germany) are both helpful to microencapsulate barely soluble drugs, increasing their apparent solubility, dissolution, and permeation [22,23].

As far as we know, there are no reports regarding the effect of microencapsulation, either by ionic gelation or spray drying, over the dissolution and permeation of EB across synthetic and biological membranes. Additionally, the impact of the encapsulating polymers on the apparent solubility of EB in *Salmo salar* intestinal conditions is unknown.

Therefore, the objectives of this study were (i) to study the dissolution/permeation and the release kinetics of EB microencapsulated with alginate (EB–ALG) and Soluplus^®^ (EB–SOL) using lipophilic and hydrophilic synthetic and biological membranes (proximal intestines). Two donor media were studied, an emulsion (EM) composed of a mixture of vegetable oils, and a Ringer solution, to simulate the intestinal medium of *Salmo salar*, and (ii) to estimate the amount of microencapsulated EB necessary to achieve a therapeutic dose.

## 2. Results and Discussion

**Figure 1:**
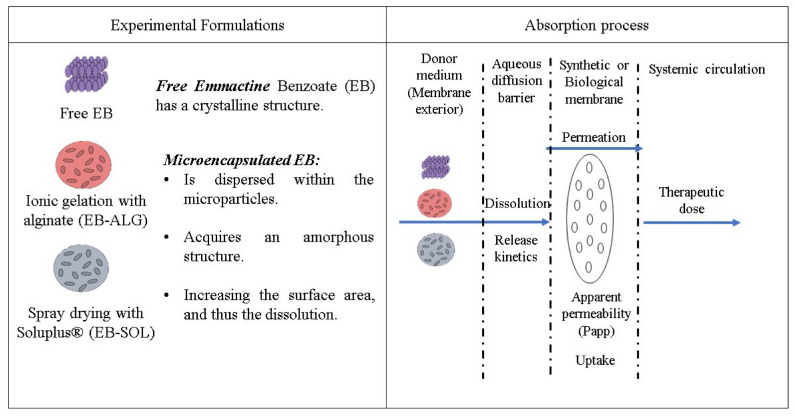
Experimental approach.

To determine the dissolution/permeation, the release mechanism, the apparent permeability coefficient, uptake, and therapeutic dose of microencapsulated emamectin benzoate (EB) were examined. Two polymers were used for the encapsulation: alginate (ALG) and Soluplus^®^ (SOL). The first used an ionic gelation encapsulation, while the second polymer used a spray drying technique. The microparticles were analyzed in both synthetic and biological membranes (Figure 1).

### 2.1. Accumulated Dissolution/Permeation of the Microencapsulated Emamectin Benzoate

In order to determine how much of the encapsulated EB crosses the intestinal membrane and becomes available for absorption, the accumulated dissolution/permeation was determined by measuring the final EB concentration after the passage through the synthetic and biological membranes.

#### 2.1.1. Accumulated Dissolution/Permeation Measured in Synthetic Membranes

**Figure 2:**
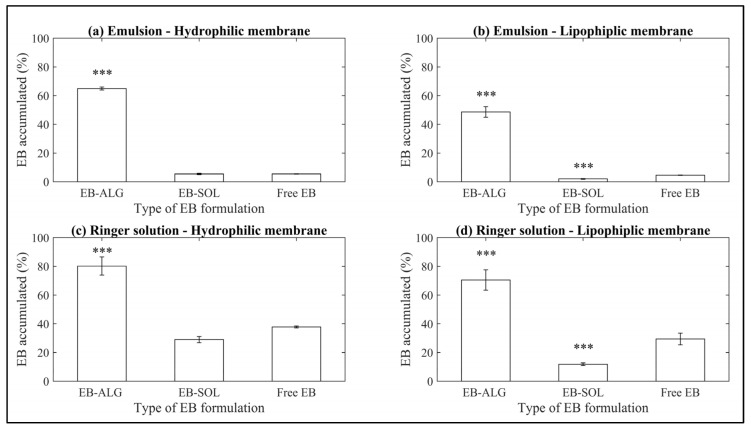
Accumulated dissolution/permeation (%) of EB using synthetic membranes. (**a**) Represents the emulsion as donor media with the hydrophilic membrane (EM–H); (**b**) represents the emulsion as donor media with the lipophilic membrane (EM–L); (**c**) represents the Ringer solution as donor media with the hydrophilic membrane (RS–H); and (**d**) represents the Ringer solution as donor media with the lipophilic membrane (RS–L). Values represent the mean with standard deviation, *n* = 3, with an initial concentration of microparticles of 0.53 mg/mL for emulsion, and 0.56 mg/mL for Ringer solution. *p* < 0.001 (***)was taken to be statistically different compared to free EB (control), calculated by a t-student test.

**Figure A1:**
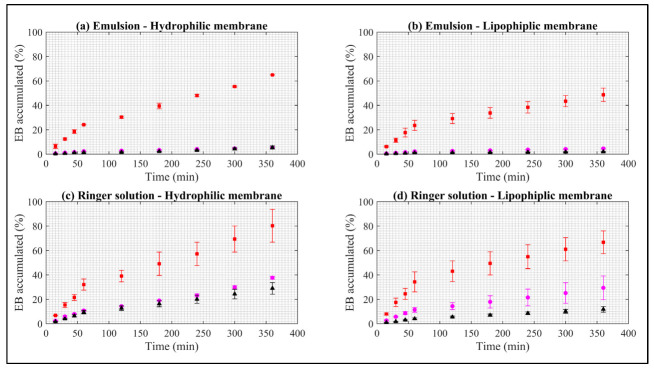
Dissolution rate of EB in synthetic membranes. Red squares represent EB–ALG microparticles, black triangles represent EB–SOL microparticles, and magenta circles represent free EB. (**a**) Represents emulsion as donor media with hydrophilic membrane. (**b**) Represents emulsion as donor media with lipophilic membrane. (**c**) Represents Ringer solution as donor media with hydrophilic membrane. (**d**) Represents Ringer solution as donor media with lipophilic membrane. Values represent the mean with standard deviation, *n* = 3.

For all the treatment conditions (type of donor media and membrane), the microparticles formed by ionic gelation show the highest accumulated dissolution and permeation (Figure 2). The dissolution/permeation profiles over time can be seen in Appendix A, Figure A1.

In descending order for EB–ALG, the highest EB concentration achieved is by RS–H with 0.452 mg/mL, followed by RS–L with 0.376 mg/mL, EM–H with 0.346 mg/mL, and EM–L with 0.259 mg/mL. These values represent 80%, 66.7%, 64.9%, and 48.6% of the EB microencapsulated, respectively. The average of EB–ALG in the EB dissolution/permeation results in the four combinations of donor media and membrane is 65% (Figure 2).

These results indicate that microencapsulation with ALG improves the accumulated dissolution/permeation of EB, mainly when the donor media is the Ringer solution, and a hydrophilic membrane is used (RS–H). In contrast, when a lipophilic donor medium or lipophilic membrane is added, as is the case with RS–L and EM–H, the dissolution/permeation is reduced by 13.3% and 15.1%, respectively, compared to the concentration of EB reached in RS–H. However, even with the lowest value of EB–ALG (EM–L), the results are superior to free EB. This result could be associated with the hydrophilic bonds or interactions formed by ionic gelation within the EB and ALG.

Sodium alginate forms hydrogels in the presence of calcium ions. Hydrogels are network structures of a hydrophilic nature, due to hydrophilic groups, such as free hydroxyl (-OH), carboxyl (-COOH), and hydrogen receptors, such as O- [24]. At the same time, emamectin benzoate also has -OH groups, indicating that both molecules could be hydrogen donors or acceptors, forming hydrogen bonds favoring the hydrophilic affinity of EB.

On the other hand, alginate can also acquire an amphiphilic nature (hydrophilic and hydrophobic), by creating hydrophobic bonds. Hydrophobic bonds are reversible noncovalent interactions. The alginate skeleton might create covalent bonds with hydrophobic residues (i.e., long alkyl chains or aromatic groups) present in emamectin benzoate. These types of hydrophobic interactions are potential vehicles for EB [24]. At the same time, hydrophilic interactions facilitate the solubility of the polymer and EB in water. Therefore, the ionic gelation process allows the formation of different structures, with regions rich in hydrophilic and hydrophobic content, in the EB microparticles.

In contrast, the microparticles formed by spray drying with SOL show significantly lower accumulated dissolution/permeation values. In descending order, the highest EB concentration achieved is for RS–H with 0.163 mg/mL, followed by RS–L with 0.066 mg/mL, EM–H with 0.029 mg/mL, and EM–L with 0.01 mg/mL. These values are 28.9%, 11.8%, 5.44%, and 1.98% of the EB microencapsulated, respectively. The average of EB–SOL in the EB dissolution/permeation results in the four combinations of donor media and membrane is 12% (Figure 2). Moreover, compared with the free EB, the EB–SOL microparticles generally show lower accumulated dissolution/permeation values (Figure 2). The values observed in the Ringer solution for the hydrophilic membrane (0.163 mg/mL, 28.9%) and lipophilic membrane (0.06 mg/mL, 11.8%) are even lower than those observed for the free drug (37.6% or 0.2 mg/mL and 29.4% or 0.16 mg/mL) for the hydrophilic and lipophilic membranes, respectively (Figure 2).

Contrary to our results, previous research shows that Soluplus^®^ increases the drug solubility [23,25,26,27], permeability [23], and absorption, using in vivo tests through pharmacokinetic studies [25,26]. However, most methods were validated according to the human release intestinal conditions, with a temperature of 37 °C and PBS or distilled water. These conditions differ from our release conditions, since we used the habitat conditions of *Salmo salar*, at a temperature of 10 ± 2 °C, and in RS-like media, which has the same PBS salts, including NaHCO_3_, CaCl_2_, MgSO_4_, glucose, and HEPES. This suggests that the presence of salts is responsible for the contradictory results. Previous research shows that the salts influence the rheological properties of Soluplus^®^. The NaCl and KCl salts generate a thermothickening effect by “salting out”, which describes a reduction in solubility due to an enhancement of the hydrophobic interaction of SOL [28]. However, a higher release is observed in the hydrophilic medium since, on average, the dissolution/permeation results in RS (20.3%) are higher than in EM (3.7%). These results indicate that salts hamper the dissolution/permeation results of EB–SOL, although with a more hydrophilic than lipophilic tendency.

Based on this interaction, microencapsulation with SOL reduces the free fraction of EB available for permeability, and due to the interaction between SOL and EB, EB cannot be absorbed. On the contrary, the synthetic membrane results show that EB–ALG represents the best option to increase the dissolution/permeation of EB.

#### 2.1.2. Dissolution/Permeation Results in Biological Membranes

**Figure 3:**
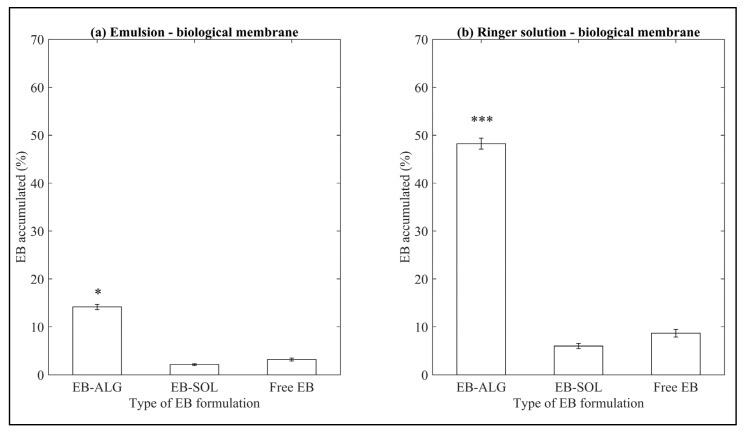
Accumulated dissolution/permeation (%) of EB determined using ex vivo biological membranes. (**a**) Represents EB accumulated in emulsion (EM) as the donor media with biological membrane, and (**b**) represents EB accumulated in Ringer solution (RS) as donor media with biological membrane. Values represent the mean with standard deviation, *n* = 5, with an initial concentration of microparticles of 0.53 mg/mL for emulsion, and 0.56 mg/mL for Ringer solution. *p* < 0.05 (*) and *p* < 0.001 (***) were taken to be statistically different compared to free EB (control), calculated by a t-student test.

**Figure A2:**
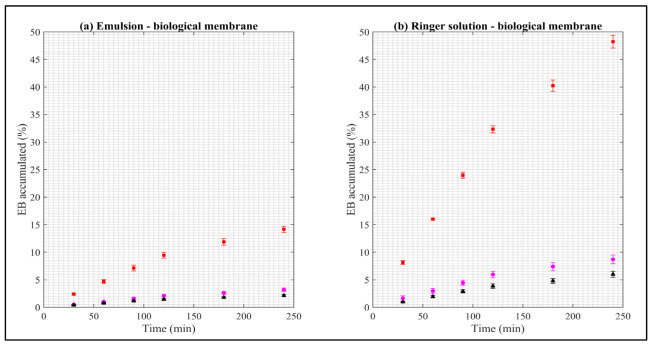
Dissolution rate of EB in biological membranes. Red squares represent EB–ALG microparticles, black triangles represent EB–SOL microparticles, and magenta circles represent free EB. (**a**) Represents emulsion as donor media with biological membrane and (**b**) Represents Ringer solution as donor media with biological membrane. Values represent the mean with standard deviation, *n* = 5.

Similar to what is observed for the synthetic membrane, when a biological membrane is used, the EB–ALG microparticles show the highest accumulated dissolution/permeation percentages in both donor media (Figure 3). The dissolution/permeation profiles over time can be seen in Appendix A, Figure A2.

These results suggest that ALG microencapsulation increases the hydrophilic affinity when using a biological membrane, consistent with the synthetic membranes’ dissolution/permeation results. However, the percentage values of dissolution/permeation using biological membranes are lower than those observed when synthetic membranes are used. In descending order, the values obtained for EB–ALG are RS–B with 0.272 mg/mL and EM–B with 0.076 mg/mL, in the biological membrane. These values represent 48.2% and 14.1% microencapsulated EB, respectively. Considering the values obtained in synthetic membranes, for RS, the average is 73.3%, which indicates a reduction of 25.1% when using the biological membrane. Similarly, for EM, the average is 56.7%, indicating a 42.6% reduction when using the biological membrane.

Similar to what is observed in synthetic membranes, the dissolution/permeation results of the EB–SOL microparticles are significantly lower than the EB–ALG microparticles and free EB (Figure 3). In descending order, the values are RS with 0.034 mg/mL, followed by EM with 0.011 mg/mL, which represent 6% and 2.13% of the EB microencapsulated, respectively. Although a higher value is obtained in RS than in EM, it does not exceed the value obtained by free EB in both donor media. This value indicates an interference of the salts in the polymer by swelling, which is reflected in the low amount of permeated EB. When comparing the results with the synthetic membrane, the EB value obtained with RS as a donor medium, using the biological membrane, results in a lower value (0.03 mg/mL) than the average EB obtained in the same donor medium with a synthetic membrane (0.11 mg/mL). On the other hand, for emulsion, the EB value obtained using the biological membrane results in an even lower value (0.011 mg/mL), compared to the average EB obtained in the same donor medium with a synthetic membrane (0.019 mg/mL). This difference might be related to the biological membrane’s greater complexity compared to the synthetic membrane, since the diffusion of the drug is through transcellular and paracellular channels, which require lipophilic molecules for transport. Our results indicate that encapsulation with Soluplus^®^ is not a feasible procedure to increase the permeation and potential absorption of EB.

### 2.2. Release Mechanism of the Microparticles

The release mechanisms of the microparticles in the donor media and intestine sources were evaluated by fitting kinetics models, using the dissolution/permeation values observed for 360 min for the synthetic membrane, and 240 min for the biological membrane (Appendix A, Table A2). The number of fitting parameters and the confidence interval size were decisive in choosing the best-fit model.

#### 2.2.1. Release Mechanism in Synthetic and Biological Membranes

According to the observed data, the model that best fits the release kinetics for both donor media, with synthetic and biological membranes, is Korsmeyer–Peppas, with an R^2^ > 0.96 (Appendix A, Table A2), suggesting that the release follows an anomalous transport, and is governed by both diffusion and swelling [29,30,31].

Although we have little information about the best-fit release kinetic model of dissolution/permeation data of EB microencapsulated with ALG, previous research using the same polymer and non-soluble drugs found similar results. Voo et al. [32] study the dissolution rate of methylene blue microencapsulated with sodium alginate in sodium phosphate buffer (0.1 M, pH 7.4) as a release medium. The authors found that the best-fit model is Korsmeyer–Peppas (R^2^ ≥ 0.99). Although this research does not use the complete list of salts used in this work, the pH is similar to ours (7.8), highlighting the importance of pH in the release mechanism using alginate in the presence of any salt.

For EB–SOL microparticles, and the emulsion medium coupled with either the lipophilic membrane (EM–L) or the Ringer solution, with both membrane natures (RS_–_H and RS–L), the best-fit model is also Korsmeyer–Peppas (R^2^ > 0.96). Kulkarni and Belgamwar [33] study the release of morin hydrate (poor aqueous solubility) microencapsulated by spray drying with Soluplus^®^, and found that the model that best-fit the compound release is a Weibull (R^2^ = 0.984), and that Korsmeyer–Peppas is the second best-fitted model (R^2^ = 0.983). The Weibull method is an empirical model, while Korsmeyer–Peppas is a semi-empirical model. While Weibull is based on experimental data, Korsmeyer–Peppas is based on both theory and experimental data. Both methods are widely used in the study of drug release, and have an exponential parameter that defines the release curve. However, unlike the Weibull model, Korsmeyer–Peppas takes into account the geometry of the release to establish the values of the release exponent (n) to indicate the release mechanism [20].

Finally, for EB–SOL microparticles, a zero-order mathematical model shows the best-fitted parameters (R^2^ ≥ 0.97) when the emulsion–hydrophilic membrane (EM–H) is studied (Appendix A, Table A2a). In this condition, the EB release depends only on time, and takes place at a constant rate, independent of the concentration of EB.

#### 2.2.2. Comparison of Release Kinetic Model between Synthetic and Biological Membranes

To determine if the use of synthetic membranes correlates with the use of biological membranes, an ANOVA was applied to find statistical differences between the dissolution/permeation results between the donor–biological membrane media and the donor–synthetic membrane media. In descending order of *p*-values, the pairs that are statistically similar between the biological and synthetic membranes are EM–B and EM–L for EB–SOL (*p* = 0.698), followed by RS–B and RS–H for EB–ALG (*p* = 0.274), then RS–B and RS–L for EB–ALG (*p* = 0.247), EM–B and EM–H for EB–SOL (*p* = 0.246), and lastly, RS–B and RS–L for EB–SOL (*p* = 0.168) (Appendix A, Table A1). Meanwhile, the pairs that are not statistically similar between the biological and synthetic membranes are RS–B and RS–H for EB–SOL (*p* = 0.020), followed by EM–B and EM–H for EB–ALG, and lastly, EM–B and EM–L for EB–ALG (*p* = 0.007).

**Figure 4:**
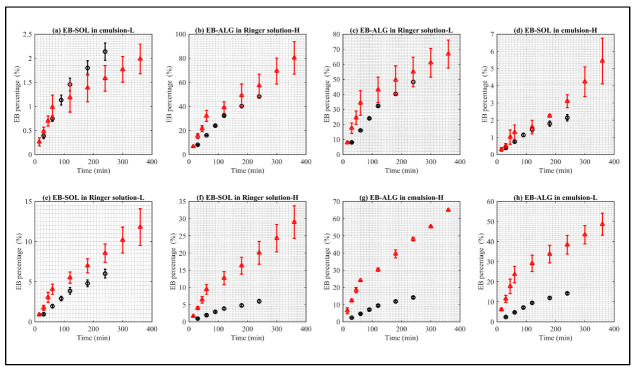
Comparison between the EB dissolution/permeation results in synthetic membranes (red points) and biological membranes (black points). (**a**) Represents EB–SOL in emulsion as donor media with lipophilic membrane (EM–L), (**b**) represents EB–ALG in Ringer solution as donor media with hydrophilic membrane (RS–H), (**c**) represents EB–ALG in Ringer solution as donor media with lipophilic membrane (RS–L), (**d**) represents EB–SOL in emulsion as donor media with hydrophilic membrane (EM–H), (**e**) represents EB–SOL in Ringer solution as donor media with lipophilic membrane (RS–L), (**f**) represents EB–SOL in Ringer solution as donor media with hydrophilic membrane (RS–H), (**g**) represents EB–ALG in emulsion as donor media with hydrophilic membrane (EM–H), and (**h**) represents EB–ALG in emulsion as donor media with lipophilic membrane (EM–L). Values represent the mean with standard deviation, *n* = 3 for FDC, and *n* = 5 for UCh.

In Figure 4, statistically similar dissolution/permeation profiles are shown in the letters a–e, while letters f–h have statistically different profiles.

The release rate constant (k) indicates the rate at which EB crosses the membrane, while the Korsmeyer–Peppas release exponent (n) indicates the release mechanism, through either Fickian or non-Fickian diffusion. In non-Fickian diffusion, there are the following mechanisms: anomalous transport, Case I transport, and Super Case II transport [20]. Fick diffusion is characterized by a high rate of solvent diffusion into the polymer matrix and a low rate of polymer relaxation, while in non-Fickian mechanisms, the main difference is the rate of diffusion of the solvent (Vs) and the relaxation rate of polymer (Vr). In Case I, Vs < Vr; in anomalous transport, Vs = Vr; and in Super Case II, Vs > Vr [20].

The release rate constant (k) of the statistically similar curves between the biological and the synthetic membranes, according to the descending order of Figure 4, are: (i) EB–SOL, in the EM–biological (4^−4^) and EM–L synthetic (1.0^−3^); (ii) EB–ALG in the RS–biological (7.7^−3^) and RS–H synthetic (1.9^−2^); (iii) EB–ALG in the RS–biological (7.7^−3^) and RS–L synthetic (3.8^−2^); (iv) EB–SOL, in the EM–biological (4^−4^) and EM–H synthetic (1.4^−4^); and (v) EB–SOL, in the RS–biological (8^−4^) and RS–L synthetic (2.8^−3^). (See Appendix A, Table A2).

The release rate constant (k) of the statistically different curves between the biological and the synthetic membranes, according to the descending order of Figure 4, are: (i) EB–SOL, in the RS–biological (8^−4^), is different from RS–H synthetic (3.7^−3^); (ii) EB–ALG, in the EM–biological (2.3^−3^), is different from EM–H synthetic (1.5^−2^); and (iii) EB–ALG, in the EM–biological (2.3^−3^), is different from EM–L synthetic (2.4^−2^) (See Appendix A, Table A2).

The results of both formulations indicate the same hydrophilic nature for the EB–ALG microparticles, and a lipophilic nature for the EB–SOL microparticles in both the donor medium and the membrane; the velocity rates have the most significant similarity. Instead, they have the biggest difference when the same lipophilic nature is used for the EB–ALG microparticles. The same behavior is observed when a hydrophilic nature is used for the EB–SOL microparticles. This result suggests that similar velocity rates between the biological and synthetic membrane are achieved using the appropriate combination of donor medium and membrane.

The release exponent (n) for EB–ALG in the biological membrane for EM and RS indicate that the release mechanism is diffusion and swelling simultaneously (anomalous transport), with 0.75 < n < 0.76, which is in the theoretical range of this type of mechanism (0.43 < n < 0.85). The anomalous transport is observed with EM–H and RS–H in the synthetic membrane, with 0.48 < n < 0.63. This result suggests that, for EB–ALG, the determination of dissolution/permeation using synthetic and biological membranes estimates a similar release mechanism for the hydrophilic (H) membrane. This similarity does not mean that the proximal intestine has a hydrophilic nature, but rather that the dissolution/permeation mechanism obtained with the formulations has better results in membranes with hydrophilic characteristics.

Similarly, for EB–SOL, the release exponent (n) in the biological membrane indicates that the release mechanism is composed of diffusion and swelling (anomalous transport), with 0.72 < n < 0.78, which is the same mechanism obtained in EM–L and RS–H using synthetic membranes, with 0.50 < n < 0.73. This result suggests that a lipophilic and hydrophilic membrane would estimate the dissolution/permeation mechanism in EM and RS in the biological membrane, respectively.

The obtained similarities indicate that it is possible to predict the dissolution/permeation behavior in the membrane similar to the proximal intestine, using only a synthetic membrane with the correct nature.

### 2.3. Apparent Permeability Coefficient

To determine if the microencapsulation is a feasible technique for EB delivery and permeation, we considered the apparent permeability coefficient (Papp), which evaluates EB permeation through the synthetic and biological membrane. Papp is distinguished from dissolution/permeation results because it also considers the volume of the receptor medium, the exposed area of the membrane, and the initial concentration of EB in the donor compartment.

#### 2.3.1. Apparent Permeability Coefficient in Synthetic Membrane

**Figure 5:**
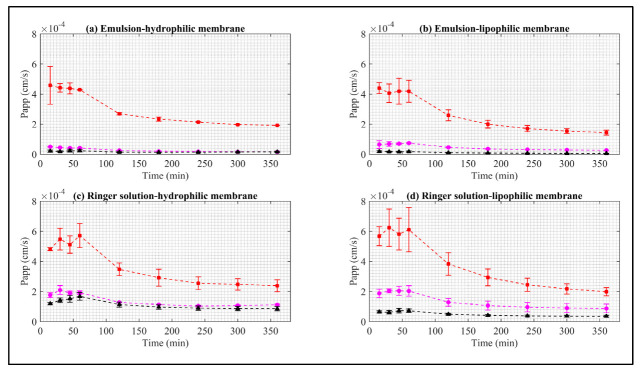
Apparent permeability of EB in synthetic membrane. Red squares represent EB–ALG microparticles, black triangles represent EB–SOL microparticles, and magenta circles represent free EB. (**a**) Represents emulsion as donor media with hydrophilic membrane (EM–H). (**b**) Represents emulsion as donor media with lipophilic membrane (EM–L). (**c**) Represents Ringer solution as donor media with hydrophilic membrane (RS–H). (**d**) Represents Ringer solution as donor media with lipophilic membrane (RS–L). Values represent the mean with standard deviation (*n* = 3).

The Papp curves of the synthetic membrane are shown in Figure 5.

The Papp values of the EB–ALG microparticles are higher than both EB–SOL and free EB. The maximum Papp values are RS–L with 6.2 mg/mL, followed by RS–H with 5.7 mg/mL, EM–H with 4.5 mg/mL, and EM–L concentrate 4.3 mg/mL. After reaching these maximum values, Papp decreases, indicating that Papp occurs more slowly, and drug administration is reduced. In addition, the area under the Papp curve (AUC) is estimated with the same descending order with the following values: 7.1 cm∙min/s, 6.9 cm∙min/s, 5.5 cm∙min/s, and 4.9 cm∙min/s (Table 1).

Consistent with the dissolution/permeation results, Papp indicates that the highest permeability is obtained with RS as the donor medium, but with a slight preference for the lipophilic membrane. This preference demonstrates the formation of hydrophilic and lipophilic bonds between ALG and EB during the ionic gelation microencapsulation process, allowing adequate dissolution in RS, but with a significant Papp through both the lipophilic and hydrophilic membranes. Additionally, Papp maintains a sustained value between 15–60 min for the four cases, indicating that the most significant amount of EB is administered almost constantly during this time.

In contrast, for the EB–SOL microparticles, the highest Papp values, in descending order, are RS–H with 1.67 mg/mL, followed by RS–L with 0.72 mg/mL, EM–H with 0.24 mg/mL, and EM–L with 0.18 mg/mL. After reaching these values, Papp remains almost constant, except for RS–H, where a decrease is observed after these values. In addition, the area under the Papp curve is estimated, obtaining the same descending order with the following values: 2.2 cm∙min/s, 0.96 cm∙min/s, 0.33 cm∙min/s, and 0.20 cm∙min/s (Table 1). Consistent with the dissolution/permeation results, Papp indicates that the highest permeability is obtained with RS as the donor medium, but with a significant preference for the hydrophilic membrane. This result suggests the selection of EB–SOL for the hydrophilic nature since the highest Papp occurred in this nature. In addition, for RS–L, EM–H, and EM–L, Papp maintains a sustained value between 15–360 min, indicating that the most significant amount of EB is administered almost constantly during this time. For RS–H, it presents a ramp until reaching 60 min, and then decreases until it is constant.

The maximum Papp ranges obtained for EB–ALG are 4.3^−4^–6.2^−4^ cm/s, for EB–SOL, 0.18^−4^–1.67^−4^ cm/s, and for EB free, 0.5^−4^–2.09^−4^ cm/s. The Papp results in our research are higher than those reported in previous research. Sundell et al. [34] study the Papp of 14C-mannitol in Atlantic salmon intestine segments, using the Ussing chamber in Ringer’s solution at T = 10 °C, and report a Papp ranging between 0.8^−6^–1.5^−6^ cm/s. This difference could be because the aqueous solubility of 14C-mannitol is higher than EB (0.012 mg/mL), which reduces Papp since the nature of the membrane is lipophilic. In another study, Minghetti et al. [35] study the Papp of the fluorescent molecules Lucifer yellow and dextran in the intestine of the rainbow trout (*Oncorhynchus mykiss*) in RTgutGC cell line assays. The authors report Papp ranging between 1.1^−9^ cm/s and 4.2^−6^ cm/s. In this case, the different values might be associated with the molecular size of the studied molecule, since as the molecular size increases, the Papp decreases. Schep et al. [36] obtain the Papp of fluorescein and 14C-mannitol in the intestine of salmon (*Oncorhynchus tshawytscha*), obtaining ranges between 3.3^−6^–13.1^−6^ cm/s for fluorescein, and 38.1^−6^–495^−6^ cm/s for mannitol. Schug et al. [37] obtain the Papp of damascone beta in the intestinal cell line of rainbow trout (*Oncorhynchus mykiss*), using RTgutGC, recording a Papp of 6.1^−5^ cm/s through the layer cellular. Sundell and Sundh [38] obtain the Papp of cortisol in the intestine of rainbow trout using Ussing chambers, with a Papp of 2^−6^–7^−6^ cm/s. Interestingly, in this study, a difference of 8^−6^ cm/s is obtained between the Papp in fresh water and seawater, which indicates that salts affect Papp.

#### 2.3.2. Apparent Permeability Coefficient in Biological Membranes

**Figure 6:**
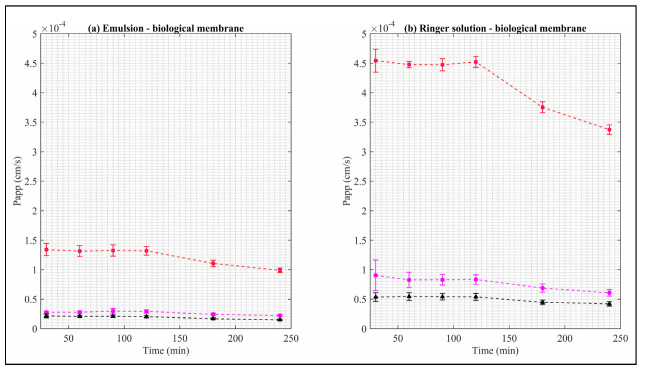
Apparent permeability of EB in the biological membrane. Red squares represent EB–ALG microparticles, black triangles represent EB–SOL microparticles, and magenta circles represent free EB. (**a**) Represents the emulsion as donor media with biological membrane, and (**b**) represents the Ringer solution as donor media with biological membrane. Values represent the mean with standard deviation (*n* = 5).

The Papp curves when the biological membrane is used with the both donor media are shown in Figure 6.

The Papp values of the EB–ALG microparticles are higher than both EB–SOL and free EB. The maximum Papp values are RS–B with 4.54 cm/s, followed by EM–B with 1.34 cm/s. Upon reaching these maximum values, Papp decreases. In addition, the area under the Papp curve is estimated, obtaining the same descending order, with the following values: 5.19 cm∙min/s and 1.52 cm∙min/s, respectively (Table 2).

The Papp and AUC values of EB–ALG microparticles indicate that microencapsulation with alginate overcomes the biological membrane’s complexity, and demonstrates that the microparticles possess both hydrophilic and lipophilic affinity, since the Papp in RS–B is higher than EM–B. The Papp values are consistent with the dissolution/permeation results, which have higher RS–B values than EM–B. Additionally, for RS–B, Papp maintains an almost constant value from 30–120 min, indicating that a significant amount of EB is permeated in this time.

The Papp for the EB–SOL microparticles in the biological membrane shows lower values than free EB, indicating that EB is trapped in the microparticle and, therefore, the microencapsulation with Soluplus^®^ using spray drying does not improve the permeation. In contrast, the EB–ALG microparticles show better Papp results compared to free EB.

#### 2.3.3. Comparison of the Apparent Permeability Coefficient between Synthetic Membranes and Biological Membranes

**Figure 7:**
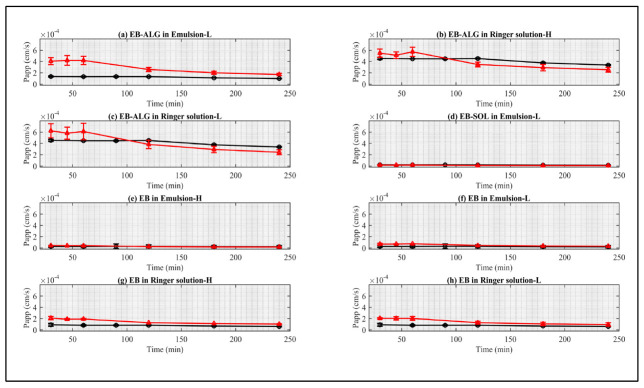
Comparison of Papp (cm/s) of synthetic membranes (red points) and biological membranes (black points). (**a**) Represents EB–ALG in emulsion as donor media with lipophilic membrane (EM–L) for FDC; (**b**) represents EB–ALG in Ringer solution as donor media with hydrophilic membrane (RS–H) for FDC; (**c**) represents EB–ALG in Ringer solution as donor media with lipophilic membrane (RS–L); (**d**) EB–SOL in emulsion as donor media with lipophilic membrane (EM–L) for FDC; (**e**) EB in emulsion with hydrophilic membrane (EM–H); (**f**) EB in emulsion as donor media with lipophilic membrane (EM–L); (**g**) EB in Ringer solution as donor media with hydrophilic membrane (RS–H); and (**h**) EB in Ringer solution as donor media with lipophilic membrane (RS–L). Values represent the mean with standard deviation, *n* = 3 for synthetic membrane and *n* = 5 for biological membrane.

To determine if using synthetic membranes instead of proximal intestines as biological membranes to estimate Papp in *Salmo salar* is feasible, RS–B is compared with RS–H and RS–L, and EM–B with EM–H and EM–L for the formulations EB–ALG, EB–SOL microparticles, and free EB. The statistically similar curves (*p* > 0.05) are shown in Figure 7.

Papp curves that are statistically similar between the biological membrane and the synthetic membrane are observed for the EB–ALG microparticles in EM–L, RS–H, and RS–L; and EB–SOL in EM–L. For free EB, significant similarities are observed for EM–H, EM–L, RS–H, and RS–L. Interestingly, the EB–ALG microparticles that result in higher dissolution/permeation and Papp values are feasible for replacing biological membranes with synthetic ones, when suitable nature conditions are present. On the other hand, the EB–SOL microparticles only allow replacement in EM–L. Finally, for free EB, it is possible to replace the biological membranes in the four combinations of the donor medium-nature with synthetic membranes.

Previous research shows similarities between synthetic membranes and biological membranes. Kaur et al. [39] compare the permeation between the synthetic membrane Strat-MTM in rat, human, and porcine ear (biologic membrane) in nanoformulations of amphotericin B, and the results indicate that Strat-MTM shows similar results to human skin. Carrer et al. [40] study the permeation of lidocaine, diclofenac sodium, and betamethasone dipropionate using synthetic membranes, using lanolin in FDC and a skin-like biological membrane. The results indicate that the three substances with the synthetic membranes have a similar absorption to that of skin.

Our Papp results suggest that using the synthetic membrane can mimic the Papp of the proximal intestine as a biological membrane, using the appropriate nature.

### 2.4. Emamectin Benzoate Uptake in the Biological Membrane

**Figure 8:**
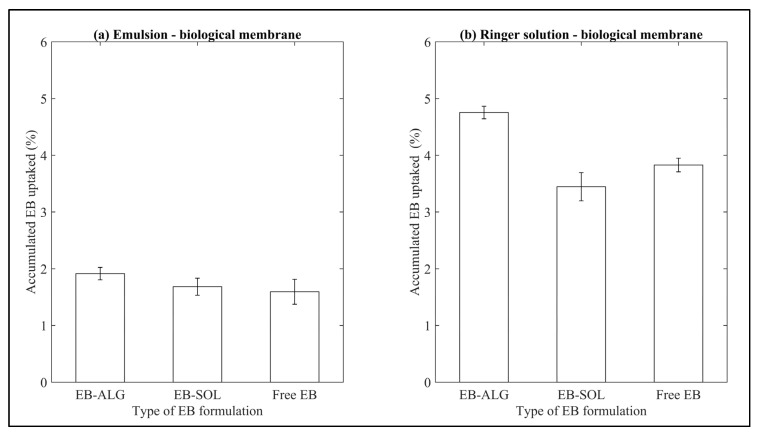
Results of accumulated EB uptake in biological membranes. (**a**) Represents the EB uptake in emulsion as donor media, and (**b**) represents the EB uptake in Ringer solution as donor media. Values represent the mean with standard deviation of five replicates.

The uptake for EB–ALG and EB–SOL microparticles, and free EB for both emulsion and Ringer’s solution as donor media can be seen in Figure 8.

The uptake for the EB–ALG and EB–SOL microparticles, and free EB is higher in RS (4.0 ± 0.6%), than in EM (1.7 ± 0.1%). In the case of RS, the EB–ALG microparticles show the highest uptake (4.7%), followed by free EB (3.8%), then the EB–SOL microparticles (3.4%). In EM, the EB–ALG microparticles show the highest uptake (1.9%), followed by EB–SOL and free EB (1.6%) (Figure 8). The accumulated concentration of EB uptake in the biological membrane is shown in Table 3.

When comparing these results with the ones observed for the dissolution/permeation, it is possible to observe whether the uptake is a facilitator or limiter of the EB absorption process.

By adding the amount of EB dissolute/permeate and the uptake, the total amount of EB available for absorption (total EB) is obtained. With value, the percentage of uptake is calculated by dividing it by the total EB. This value is then used to determine if the uptake is a facilitator or limiter in the absorption of EB. A facilitating uptake should result in low amounts of EB, while if it is a limiter, it presents high amounts of EB (i.e., the amount of EB that remains in the membrane and does not permeate).

The ascending order of uptake values for RS is EB–ALG with 7.3%, free EB with 35.3%, and EB–SOL with 36.4%. These results suggest that the uptake is a permeation facilitator for the EB–ALG microparticles. That is to say, the affinity between the EB microencapsulated with alginate and the biological membrane allows a suitable passive transport of EB. For free EB and EB–SOL microparticles, uptake is a limitation, due to the high amount of EB in the uptake site.

In the case of EM, in ascending order, the highest values are the EB–ALG microparticles with 10.1%, followed by free EB with 37.5%, and finally, the EB–SOL microparticles with 44.1%. Similarly, in RS as donor media, the EB–ALG microparticles obtain a low amount of absorption; so in this case, the uptake is a facilitator, while for free EB and the EB–SOL microparticles, uptake is a limiter for absorption.

The previous results indicate that the uptake of the EB–ALG microparticles, unlike the EB–SOL microparticles and free EB, is low due to the effectiveness of EB transport through the membrane, which is based on electrochemical gradients such as Na+, K+, and ATPase [41].

### 2.5. Apparent Solubility (Sapp) of Emamectin Benzoate in Presence of Sodium Alginate and Soluplus^®^

The apparent solubility indicates the drug solubility empirically determined in a specific solvent system. In our research, the release media have specific conditions, such as salts compositions and oils that comprise the *Salmo salar* food. In that sense, estimating the apparent solubility of EB in the presence of Soluplus^®^ and sodium alginate is essential for this specific release medium. Different ratios of EB: polymer are used to find the effect in the apparent solubility (Sapp) of alginate and Soluplus^®^ on EB. The ratios of EB: polymer are 1:0, 1:1, 1:3, 1:5, 1:7, and 1:9 with initial EB concentrations of 40, 20, 13.3, 8.0, 5.7, and 4.4 mg/mL, respectively, in a total amount of 40 mg/mL with the polymer.

The highest percentages of solubilized EB are obtained in the following descending order: the EB–ALG microparticles in EM (53.1%, 2.36 mg/mL), followed by the EB–SOL microparticles in EM (35.4%, 2.02 mg/mL), the EB–SOL microparticles in SR (7.08%, 0.314 mg/mL), and the EB–ALG microparticles in SR (1.64%, 7.29^−2^ mg/mL) (Table 4).

The average percentage of EB solubilized in EM (23.4%) is higher than in RS (1.99%), which differs from the dissolution/permeation results where the EB–ALG and EB–SOL microparticles result in higher amounts for EB in RS–H. This difference is likely to occur because the Sapp is obtained from the drug and the polymer mixture. However, it does not pass through the microencapsulation process, which involves the creation of new interactions and bonds that probably change the preference for a hydrophilic nature. Despite the differences, the data acquired show that, even with a lower amount of polymer, the Sapp improves, indicating that the use of both polymers increases the solubility of EB. This increase makes it possible to reduce the amount of EB administered in order to achieve the desired dose.

### 2.6. Determination of the Therapeutic Dosage, Enclosed in the Microparticles, of Emamectin Benzoate for Atlantic Salmon

Microencapsulation with ALG is the only formulation that improves the dissolution/permeation of free EB in the biological membrane. Therefore, the dosage is calculated only for this formulation (Table 5).

The current therapeutic dose of free EB to control *C. rogercresseyi* is 0.05 mg/kg of biomass for seven days, for a 0.1 kg fish, with a blood volume of 4 mL, and, therefore, in order to achieve the therapeutic amount, a dose of 1.25^−3^ mg/mL of EB is required. According to the results in the EM medium with the EB–ALG microparticles, in order to achieve the dosage, 8.8^−3^ mg/mL of encapsulated EB is required; while if free EB is used, the amount increases to 3.9^−2^ mg/mL. Therefore, the use of EB–ALG microparticles reduces the amount of EB needed to achieve the therapeutic dose by 3.0^−2^ mg/mL EB. Similarly, in the RS medium, for the same microparticles, 2.6^−3^ mg of encapsulated EB is required, while in the same medium, 1.4^−2^ mg/mL of free EB is required. This results in a decrease in 1.1^−2^ mg/mL of EB necessary for the drug administration at the correct dose.

Therefore, our results indicate that when EB is encapsulated in alginate, there is a significant decrease in the amount of EB necessary in order to reach the therapeutic dose relative to free EB, especially when the delivery medium is lipophilic.

## 3. Materials and Methods

### 3.1. Reagents and Solutions

EB (90% purity) was obtained from Toronto Research Chemicals Inc (Toronto, Canada). It was microencapsulated by ionic gelation with sodium alginate (CAS:9005-38-3, Sigma-Aldrich, St Louis, MO, USA), with a ratio of EB:ALG of 1:1.1, using a pressurized sprinkler system (Colaco, Chile) with pressurized atomization (1.5 bars) through a syringe with a nozzle (Bete Fog Nozzle ¼ XA, Greenfield, MA, USA). The resulting microparticles had a load capacity of 31.1%. In addition, EB was microencapsulated by spray drying using Soluplus^®^ as the polymer with a ratio of EB:Soluplus^®^ of 1.6:1. The encapsulation was performed in a Büchi mini spray dryer B-290 (Büchi Labortechnik AG, Flawil, Switzerland), and the microparticles obtained had a load capacity 32.2%.

The Ringer’s solution used in this study was composed of 140 mM NaCl, 2.5 mM KCl, 15 mM NaHCO_3_, 1.5 mM CaCl_2_, 1 mM KH_2_PO_4_, 0.8 mM MgSO_4_, 10 mM glucose, and 5 mM HEPES buffer, with a pH of 7.8 [42]. Acetone, acetonitrile, methanol, ethanol, and phosphoric acid were purchased from Merck (Darmstadt, Germany). All organic solvents were of high-performance liquid chromatography (HPLC) grade. Emulsions were composed of 5% *w*/*v* oil mix, provided by Ewos Fish Health Center (raps oil, canola oil, linseed oil, chicken fat, and additives, Tween 20 (0.35% *w*/*v*); and Ringer’s solution with free EB, EB–SOL, or EB–ALG microparticles (94.64% *w*/*v*). The mix was stirred for 30 s in an Ultraturrax^®^ IKA T18 Basic (Campinas, Brazil), in order to obtain an emulsion.

### 3.2. Preparation of the Microparticles

EB microparticles were prepared by ionic gelation and spray drying using sodium alginate and Soluplus^®^, respectively.

In previous research, we studied the optimization of the microencapsulation conditions that were considered for the preparation of microparticles (for both ionic gelation and spray drying), including the study of optimizing yield (Y), encapsulation efficiency (EE), loading capacity (LC), as well as in vitro EB release in gastric digestion (GD), and EB release in intestinal digestion (ID). The experimental points and model of the responses were defined using a design of experiments and response surface methodology. Moreover, the suitable encapsulation parameters were estimated using three multi-criteria optimization approaches, desirability function, TSEMO + TOPSIS, and weighting method for SD; and the first two techniques were used for IG. This manuscript is currently under review (data not shown). The parameters used for this study correspond to the optimized values obtained.

#### 3.2.1. Spray Drying Encapsulation (EB–SOL)

A total of 3.16 g of EB was dissolved in 15 mL of ethanol (99%), and 1.95 g of Soluplus^®^ was dissolved in 135 mL of distilled water; then, both solutions were mixed and stirred at 40 °C for 72 h. The solution was fed into a Büchi mini spray dryer B-290 (Büchi Labortechnik AG, Flawil, Switzerland), with fixed conditions: inlet air temperature 110 °C; airflow 742 l/h; atomization pressure 1.45 psi. The feeding flows were set up at 4.8 mL/min. Outlet air temperatures observed were in the range of 60–70 °C. The spray-dried samples were stored in Falcon tubes at room temperature. The microparticles obtained the following microencapsulation characteristics: yield (Y = 65.8), encapsulation efficiency (EE = 79.2), loading capacity (LC = 32.2).

#### 3.2.2. Ionic Gelation Encapsulation (EB–ALG)

A total of 0.78 g of EB and 0.86 g of sodium alginate were weighed and prepared in 60 mL of distilled water. Then, the solution was stirred for 6 h in a multitube vortex mixer (Heidolph, Madrid, Spain), and atomized in a jetting system with pressurized atomization (1.5 bars). The solution was passed through a syringe containing a nozzle with a hole size of 2 mm (Bete Fog Nozzle ¼ XA, Greenfield, MA, USA), dropped into a CaCl_2_ solution (0.25 M) and stirred at room temperature (18 ± 1 °C), at a dropping rate of 227.3 mL/h, forming microparticles loaded with EB. The microparticles were kept in a gelling bath overnight to achieve sedimentation at room temperature, and then stored at 4 °C. The microparticles obtained the following microencapsulation characteristics: yield (Y = 85.1), encapsulation efficiency (EE = 74.1), loading capacity (LC = 31.1).

### 3.3. Dissolution/Permeation Conditions of Microencapsulated Emamectin Benzoate

#### 3.3.1. Dissolution/Permeation Conditions in Synthetic Membranes

Dissolution/permeation studies were carried out in Franz diffusion cells (Laraspiral, Dijon, France) with a permeation area of 0.5 cm^2^. The receptor compartment containing 5 mL of distilled water (pH = 7.0) was continuously stirred at 300 rpm in multiple magnetic stirrers (Velp, La Rioja, Spain). The whole system was kept at constant temperature (10 ± 2 °C) through thermostatic bath circulation. Hydrophilic and lipophilic cellulose acetate membranes, with a porosity of 0.45 µm (Sartorius, NY, USA), were used in each trial. These membranes were submerged in distilled water (hydrophilic) and octanol (lipophilic) for 12 h before the experiments. Then, each membrane was carefully placed at the interface between the donor and receptor compartments, while the two donor media used were Ringer’s solution or the *o*/*w* emulsion. Combinations of both variables were studied, according to Table 6.

EB–ALG and EB–SOL microparticles, and free EB with the same concentration (control) were added in the donor media. Aliquots of 500 µL were collected at 15, 30, 45, 60, 120, 180, 240, and 360 min by triplicate. Sink conditions were maintained with the replacement of the same volume of receptor medium. All collected samples were analyzed by HPLC (Merck, Burladingen, Germany). The equipment consisted of a DAD detector Elite LaChrome L-2455 (Merck, Burladingen, Germany), set at 243 nm. The pump used was Elite LaChrome L-2130 (Merck-Hitachi, La Rioja, Spain). The column used was Chromolith RP-18, end-capped with a length of 100 mm, an internal diameter of 4.6 mm, and a particle size of 3.5 µm (Merck, Darmstadt, Germany), at room temperature. The mobile phase was composed of acetonitrile and phosphoric acid (0.1%) 50:50, at a 1 mL/min flow rate. The quantification of EB released was obtained by the regression equation obtained from a standard curve prepared previously (0.004–0.2 mg/mL, R^2^ > 0.992).

#### 3.3.2. Dissolution/Permeation Conditions in Biological Membranes

Juveniles of Atlantic salmon, *Salmo salar,* with an average weight of 100 g were obtained from a local fish farm (Valdivia, Region de Los Rios, Chile), and kept in the Salmon Clinical Trials Facility at the Universidad Austral in Valdivia (Chile), following the welfare protocols described in the project INNOVA CORFO 09MCSS-6730, and approved by the ethics committee at the University. Fish were maintained in a recirculated system with a stocking density of 40 fish per tank (300 L each), at constant temperature and salinity (13 °C, 18 ppt) and oxygen concentration above 9 ppm. The photoperiod was 18 h light/6 h dark. Dissolved oxygen, pH, nitrite, nitrate, and ammonia were monitored throughout the experiment. The Ussing chamber system, equipped with intestinal tissues from Atlantic salmon, was prepared according to the method reported previously by Jutfelt et al. [25]. For that, fish were euthanized with an overdose of MS-222 (tricaine methane-sulfonate, 5 mL/10 L water, Sigma, St Louis, MO, USA), and the intestine proximal portion was dissected, and transported immediately into pools with a PBS buffer. Then, intestines were carefully mounted on flat sheets between two halves of acrylic chambers (exposed area = 0.3 cm^2^) in the UCh. The donor and receiver chambers were tightly screwed. The intestine’s apical and serosal sides were inserted in the donor media and the receiver medium, respectively. The entire assembly was kept in a water bath circulating system to maintain the temperature inside the chambers at 10 ± 2 °C. The volume in each compartment was the same (4 mL) to avoid damage caused by the bending of the tissue. The gas (O_2_) was administered continuously, at a pressure of 1 bar, to oxygenate the liquid contents and stir the liquid to ensure high convection. Forty µL (0.18 g/mL) of glucose was added, representing the carbohydrate intake present in the conventional feed of the *Salmo salar*. EB–ALG and EB–SOL microparticles, and free EB were added in the donor media according to the experimental design of Table 6. Aliquots of 100 µL were collected at times 30, 60, 90, 120, 180, and 240 min, with five replicates, and then cooled at −20 °C. Sink conditions were maintained with the replacement of the same volume of receptor medium. HPLC analyzed all collected samples, and quantification of EB was obtained by the regression equation obtained from a standard curve prepared previously (0.004–0.2 mg/mL, R^2^ > 0.992). Intestine samples were stored at −20 °C in glass containers before analysis. Free EB concentration used as a control was 0.56 mg/mL.

### 3.4. Estimation of Best Fitted-Release Kinetic Model

Data obtained from the dissolution/permeation results in the synthetic membranes and the biological membranes were used to fit six mathematical models commonly used to explain the release behavior of encapsulated compounds: (i) zero-order, (ii) first-order, (iii) Higuchi, (iv) Ritger–Peppas and Korsmeyer–Peppas, (v) Peppas–Sahlin, and (vi) Weibull model [20]. The best fit was defined by the coefficient of determination (R^2^), the adjusted coefficient of determination ($R^{2}{}_{\mathrm{adj}}$), the number of fitting parameters, and the confidence interval [43].

### 3.5. Estimation of the Apparent Permeability Coefficient

The apparent permeability coefficient (Papp) was obtained to evaluate the EB permeation through the synthetic and biological membrane, and it was determined according to:

(1) $$P_{\mathrm{app}}\left(\mathrm{cm}\cdot s^{-1}\right)=(\mathrm{dC}/\mathrm{dt})\cdot V/\left(A\cdot C_{0}\right)$$

where dC/dt is the change in concentration of EB on the receiver compartment per unit time $\left({\mathrm{mg}\;\mathrm{mL}}^{-1}{\;s}^{-1}\right)$, V is the volume of the receiver compartment (${\mathrm{cm}}^{3}$), A is the area of exposed membrane (${\mathrm{cm}}^{2}$), and $C_{0}$ is the initial concentration of EB in the donor compartment. The values of $P_{\mathrm{app}}$ were calculated between 15 and 360 min in synthetic membranes, and 30 to 240 min in biological membranes after adding EB–SOL and EB–ALG microparticles and free EB.

### 3.6. Emamectin Benzoate Uptake Conditions in Intestines

Intestine samples were cut into small pieces and homogenized using a mortar and pestle. Then, 1–3 g of sample homogenate was extracted with methanol and aqueous buffer (1:1). Approximately 1 mL of the extract was passed through 0.45 mm syringe filters of nylon (Fisher, Waltham, Massachusetts, MA, USA) into HPLC vials for analysis. The concentration of EB was based on liquid chromatography–tandem mass spectroscopy (LC–MS/MS). Analysis was carried out with tandem mass spectrometry (Quest Labs, Jersey, NJ, USA). The mass spectrometer had an ionization source where the LC column effluent was nebulized, desolvated, and ionized, creating charged particles. These charged particles migrate under a high vacuum through a series of mass analyzers by applying electromagnetic fields, according to Ikonomou and Surridge [44].

### 3.7. Emamectin Benzoate Apparent Solubility Conditions in Presence of Sodium Alginate and Soluplus^®^

The apparent solubility of EB in the presence of sodium alginate and Soluplus^®^ was estimated. An excess amount of EB was accurately weighed according to the EB: ALG and EB: SOL ratio shown in Table 7.

Solid dispersions were added to 2 mL of distilled water, forming a saturated solution. Samples were maintained under stirring in Multi Reax, level 4 (Merck, Germany), for 72 h at 10 ± 2 °C (temperature of *Salmo salar* body). The resultant solutions were filtered through 0.22 µm filters and analyzed in HPLC at 243 nm, in a column of Chromolith RP-18, end-capped with a length of 100 mm, an internal diameter of 4.6 mm, and a particle size of 3.5 µm (Merck, Darmstadt, Germany) at room temperature, according to the procedure followed by Zarmpi et al. [44]. All measurements were carried out in triplicate.

### 3.8. Statistical Analysis

The experiments were carried out in triplicate for the synthetic membranes analysis, while for the biological membranes they were conducted in five replicates. Data are expressed as mean values ± standard deviation. To find statistical differences between formulations (EB–ALG, EB–SOL, and free EB), an analysis of variance (ANOVA) was applied. Similarities in the membrane’s nature were found between synthetic membranes and biological membranes, according to the results of dissolution/permeation and apparent permeability, which were statistically analyzed with analysis of variance (ANOVA) using the Student’s *t*-test. Minitab was used for the statistical analysis, while for curve fitting, Matlab R2015a was used.

## 4. Conclusions

EB microencapsulation by ionic gelation using sodium alginate improves the dissolution/permeation, Papp, and Sapp of free EB, which implies reducing and protecting the amount of EB necessary to reach the dosage of EB in *Salmo salar*.

The EB–ALG microparticles yield the highest EB dissolution/permeation with accumulated Ringer solution and a hydrophilic membrane as donor media (0.45 mg/mL or 80.2%). This result is probably because during ionic gelation, EB and ALG create new hydrophilic and lipophilic bonds that increase hydrophilic and lipophilic affinity. The best-fitted model that predicted the release mechanism was Korsmeyer–Peppas, indicating that the release mechanism was an anomalous transport, or diffusion and swelling simultaneously. The Papp values of the EB–ALG microparticles are higher than both the EB–SOL microparticles and free EB. The maximum Papp value is for RS–B, with 4.54 cm/s. The uptake of the EB–ALG microparticles, unlike the EB–SOL microparticles and free EB, is low, due to the effectiveness of EB transport through the membrane, which is based on electrochemical gradients.

The microencapsulation with alginate reduces the dosage in 3.0 × 10^−2^ mg/mL and 1.1^−2^ mg/mL of EB in EM and RS, respectively.

Interestingly, our results show that the use of synthetic membranes mimics the compartment of biological membranes in *Salmo salar.* These results could positively impact the increasingly common trend of leaving aside the use of live animals for the testing of pharmaceutical products

On the contrary, the use of Soluplus^®^ for the encapsulation of EB results in lower dis-solution/permeation than free EB, and, therefore, polymer and spray drying are not a feasible technique to reduce the use of EB.

The use of both microencapsulation techniques provided novel information for improving EB absorption, and can be applied to any drug with low aqueous solubility for aquaculture use.

## Figures and Tables

**Table 1:** Apparent permeability results of synthetic membrane.

Donor Medium-Nature of Membrane	Formulation	Area under the Curve (AUC) (cm∙min/s)	Highest Papp Achieved (1^−4^) (cm/s)
Emulsion–hydrophilic membrane (EM–H)	EB–ALG	5.59 ± 0.22	4.58 ± 9.5^−3^ *
	EB–SOL	0.33 ± 0.07	0.24 ± 2.2^−3^
	Free EB	0.51 ± 0.02	0.50 ± 2.3^−4^
Emulsion–lipophilic membrane (EM–L)	EB–ALG	4.96 ± 0.69	4.39 ± 2.7^−3^ *
	EB–SOL	0.20 ± 0.04	0.18 ± 4.3^−4^ *
	Free EB	0.88 ± 0.05	0.74 ± 1.2^−3^
Ringer solution–hydrophilic membrane (RS–H)	EB–ALG	6.98 ± 1.02	5.71 ± 2.5^−2^ *
	EB–SOL	2.23 ± 0.33	1.67 ± 0.8^−2^
	Free EB	2.69 ± 0.12	2.09 ± 4.6^−3^
Ringer solution–lipophilic membrane (RS–L)	EB–ALG	7.16 ± 1.36	76.23 ± 1.9^−2^ *
	EB–SOL	0.96 ± 0.14	0.72 ± 3.6^−3^ *
	Free EB	0.59 ± 0.59	2.04 ± 2.0^−3^

EB–ALG: microparticles with alginate, EB–SOL: microparticles with Soluplus^®^, free EB: free emamectin benzoate. Data are average ± standard deviation (*n* = 3). *p* < 0.05 (*) was taken to be statistically different compared to free EB (control), calculated by a t-student test.

**Table 2:** Apparent permeability results in biological membrane.

Donor Medium-Nature of Membrane	Formulation	Area under the Curve (AUC) (cm∙min/s)	Highest Papp Achieved (1^−4^) (cm/s)
Emulsion–biological membrane	EB–ALG	1.52 ± 0.08	1.34 ± 1.0^−5^
	EB–SOL	0.23 ± 0.02	0.21 ± 3.2^−6^
	Free EB	0.33 ± 0.03	0.29 ± 4.2^−6^
Ringer solution–biological membrane	EB–ALG	5.19 ± 0.11	4.54 ± 1.9^−5^ *
	EB–SOL	0.62 ± 0.06	0.54 ± 6.6^−6^
	Free EB	0.96 ± 0.12	0.90 ± 2.6^−5^

EB–ALG: microparticles with alginate, EB–SOL: microparticles with Soluplus^®^, free EB: free emamectin benzoate. Data are average ± standard deviation (*n* = 5). *p* < 0.05 (*) was taken to be statistically different compared to Free EB (control), calculated by a t-student test.

**Table 3:** Accumulated EB uptake in biological membrane in Ussing chambers.

Donor Media	Type of Formulation	Concentration (mg/0.1 g of Intestine)
Emulsion	EB–ALG	0.244 ± 2.4^−2^ *
	EB–SOL	0.079 ± 9.0^−3^
	Free EB	0.066 ± 7.0^−3^
Ringer solution	EB–ALG	0.197 ± 2.1^−2^ ***
	EB–SOL	0.088 ± 1.0^−2^ *
	Free EB	0.083 ± 9.0^−3^

EB–ALG: microparticles with alginate, EB–SOL: microparticles with Soluplus^®^, and free EB: free emamectin benzoate. Data are average ± standard deviation (*n* = 5). *p* < 0.05 (*) and *p* < 0.001 (***) were taken to be statistically different compared to free EB (control), calculated by a t-student test.

**Table 4:** EB apparent solubility with sodium alginate and Soluplus^®^ in presence of Ringer solution and emulsion.

(a) Sapp of EB and ALG					
Denomination	EB: PolymerRatio	Apparent Solubility in Ringer Solution (mg/mL)	EB Solubilized in RS (%)	Apparent Solubility in Emulsion (mg/mL)	EB Solubilized in EM (%)
EB–ALG-1:0	1:0	7.16^−2^ ± 7.5^−3^	0.17	2.35 ± 0.10	5.89
EB–ALG-1:1 (diss/per)	1:1	7.35^−2^ ± 7.5^−3^	0.36	2.36 ± 0.10	11.8
EB–ALG-1:3	1:3	7.38^−2^ ± 7.3^−3^	0.55	2.35 ± 0.10	17.6
EB–ALG-1:5	1:5	7.28^−2^ ± 7.5^−3^	0.91	2.35 ± 0.10	29.4
EB–ALG-1:7	1:7	7.30^−2^ ± 7.6^−3^	1.27	2.36 ± 0.10	41.3
EB–ALG-1:9	1:9	**7.29^−2^ ± 7.4^−3^**	**1.64**	**2.36 ± 0.10**	**53.1**
(b) Sapp of EB and SOL					
EB–SOL-1:0	1:0	7.17^−2^ ± 7.5^−3^	0.17	2.35 ± 0.10	5.89
EB–SOL-1.6:1 (diss/per)	1.6:1	0.237 ± 1.6^−2^	0.89	2.71 ± 0.10	10.1
EB–SOL-1:1	1:1	0.294 ± 2.6^−2^	1.47	2.81 ± 0.18	14.0
EB–SOL-1:3	1:3	0.362 ± 1.1^−2^	2.71	2.36 ± 0.14	17.7
EB–SOL-1:5	1:5	0.333 ± 2.3^−2^	4.16	2.05 ± 0.12	25.6
EB–SOL-1:7	1:7	0.325 ± 1.4^−2^	5.69	**2.02 ± 0.19**	**35.4**
EB–SOL-1:9	1:9	**0.314 ± 2.4^−2^**	**7.08**	1.50 ± 0.04	33.7

EB: emamectin benzoate, ALG: alginate, SOL: Soluplus^®^. Numbers in bold are the best results in each donor media. Data are average ± standard deviation (*n* = 3).

**Table 5:** Amount of EB in formulations to reach the therapeutic dose against *C. rogercresseyi*.

Formulation	Amount of EB to Be Administered in Emulsion (mg/mL)	Amount of EB to Be Administered in Ringer Solution (mg/mL)
Free EB	3.9^−2^	1.4^−2^
EB–ALG	8.8^−3^	2.6^−3^

**Table 6:** Experimental design of synthetic membranes and biological membranes.

Membrane Type	Experiment Number	Donor Media	Formulation	Membrane Nature
Synthetic membrane	1	Emulsion	EB–SOL microparticles	Lipophilic
	2			Hydrophilic
	3		EB–ALG microparticles	Lipophilic
	4			Hydrophilic
	5		Free EB	Lipophilic
	6			Hydrophilic
	7	Ringer solution	EB–ALG microparticles	Lipophilic
	8			Hydrophilic
	9		EB–SOL microparticles	Lipophilic
	10			Hydrophilic
	11		Free EB	Lipophilic
	12			Hydrophilic
Biological membrane	1	Emulsion	EB–ALG microparticles	Lipophilic
	2		EB–SOL microparticles	
	3		Free EB	
	4	Ringer solution	EB–ALG microparticles	
	5		EB–SOL microparticles	
	6		Free EB	

EB–ALG: microparticles of ionic gelation, EB–SOL: microparticles of spray drying, and free EB: free emamectin benzoate.

**Table 7:** Composition of solid dispersions of Emamectin benzoate with polymers.

Denomination	Proportion EB: ALG	Denomination	Proportion EB:SOL
EB–ALG-1:0	1:0	EB–SOL-1.6:1 (Op)	1.6:1
EB–ALG-1:1 (Op)	1:1	EB–SOL-1:0	1:0
EB–ALG-1:3	1:3	EB–SOL-1:1	1:1
EB–ALG-1:5	1:5	EB–SOL-1:3	1:3
EB–ALG-1:7	1:7	EB–SOL-1:5	1:5
EB–ALG-1:9	1:9	EB–SOL-1:7	1:7
		EB–SOL-1:9	1:9

EB: emamectin benzoate, ALG: sodium alginate, SOL: Soluplus^®^, and Op: optimal ratio found by desirability function.

## Data Availability

Data is contained within the article.

## Appendix A

**Table A1:** Analysis of variance (ANOVA) between synthetic and biological membrane in the dissolution/permeation results.

Type of Formulation	Donor Media (UCh) and Donor Media—Nature of Membrane (FDC)	Accumulated EB Permeation (*p*-Value)
EB–ALG	EM (UCh) and EM–H (FDC)	0.010
	EM (UCh) and EM–L (FDC)	0.007
EB–SOL	EM (UCh) and EM–H (FDC)	0.246
	EM (UCh) and EM–L (FDC)	0.698
EB–ALG	RS (UCh) and RS–H (FDC)	0.274
	RS (UCh) and RS–L (FDC)	0.247
EB–SOL	RS (UCh) and RS–H (FDC)	0.020
	RS (UCh) and RS–L (FDC)	0.168

EB–ALG: microparticles with alginate, EB–SOL: microparticles with Soluplus^®^, free EB: free emamectin benzoate, EM: emulsion, RS: ringer solution, H: hydrophilic membrane, L: lipophilic membrane, FDC: Franz™ diffusion cell (synthetic membranes), and UCh: Ussing chambers (biological membranes).

**Table A2:** Best-fit kinetic models for EB dissolution rate.

(a) Kinetic Models in Synthetic Membrane							
Formulation	Donor Medium	Membrane Nature	Equation	Type of Model	R^2^	$R^{2}{}_{\mathrm{adj}}$	Number
EB–ALG	Emulsion	Hydrophilic	*Q* = 0.0154*t*^0.631^	Korsmeyer–Peppas	0.99	0.98	(1)
EB–ALG	Emulsion	Lipophilic	*Q* = 0.024*t*^0.506^	Korsmeyer–Peppas	0.97	0.97	(2)
EB–ALG	Ringer solution	Hydrophilic	*Q* = 0.019*t*^0.626^	Korsmeyer–Peppas	0.98	0.98	(3)
EB–ALG	Ringer solution	Lipophilic	*Q* = 0.038*t*^0.486^	Korsmeyer–Peppas	0.96	0.96	(4)
EB–SOL	Emulsion	Hydrophilic	*Q* = 1.424^−4^*t*	Zero-order	0.97	0.97	(5)
EB–SOL	Emulsion	Lipophilic	*Q* = 0.0010*t*^0.504^	Korsmeyer–Peppas	0.97	0.97	(6)
EB–SOL	Ringer solution	Hydrophilic	*Q* = 0.0037*t*^0.735^	Korsmeyer–Peppas	0.99	0.98	(7)
EB–SOL	Ringer solution	Lipophilic	*Q* = 0.0028*t*^0.5^	Korsmeyer–Peppas	0.99	0.99	(8)
(b) Kinetic models in biological membrane							
EB–ALG	Emulsion	Biological membrane	*Q* = 0.0023*t*^0.758^	Korsmeyer–Peppas	0.98	0.98	(9)
EB–ALG	Ringer solution	Biological membrane	*Q* = 0.0077*t*^0.760^	Korsmeyer–Peppas	0.98	0.98	(10)
EB–SOL	Emulsion	Biological membrane	*Q* = 0.0004*t*^0.721^	Korsmeyer–Peppas	0.98	0.98	(11)
EB–SOL	Ringer solution	Biological membrane	*Q* = 0.0008*t*^0.783^	Korsmeyer–Peppas	0.98	0.98	(12)

EB–ALG: microparticles with alginate, EB–SOL microparticles with Soluplus*^®^,* R^2^: coefficient of determination, and $R_{\mathrm{adj}}^{2}$: adjusted coefficient of determination.

**Table A3:** Analysis of variance (ANOVA) between synthetic and biological membrane in the apparent permeability results.

Type of Formulation	Donor Media (UCh) and Donor Media—Nature of Membrane (FDC)	Accumulated EB Permeation (*p*-Value)
EB–ALG	EM (UCh) and EM–H (FDC)	0.001
	EM (UCh) and EM–L (FDC)	0.008
EB–SOL	EM (UCh) and EM–H (FDC)	0.413
	EM (UCh) and EM–L (FDC)	0.009
Free EB	EM (UCh) and EM–H (FDC)	0.413
	EM (UCh) and EM–L (FDC)	0.014
EB–ALG	RS (UCh) and RS–H (FDC)	0.609
	RS (UCh) and RS–L (FDC)	0.940
EB–SOL	RS (UCh) and RS–H (FDC)	2.0^−4^
	RS (UCh) and RS–L (FDC)	0.779
Free EB	RS (UCh) and RS–H (FDC)	2.0^−4^
	RS (UCh) and RS–L (FDC)	0.010

EB–ALG: microparticles with alginate, EB–SOL: microparticles with Soluplus^®^, free EB: free emamectin benzoate, EM: emulsion, RS: ringer solution, H: hydrophilic membrane, L: lipophilic membrane, FDC: Franz™ diffusion cell, and UCh: Ussing chambers.
